# Assignment of pre-event ASA physical status classification by pre-hospital physicians: a prospective inter-rater reliability study

**DOI:** 10.1186/s12871-020-01083-x

**Published:** 2020-07-09

**Authors:** Kristin Tønsager, Marius Rehn, Andreas J. Krüger, Jo Røislien, Kjetil G. Ringdal

**Affiliations:** 1 Department of Research, The Norwegian Air Ambulance Foundation, Oslo, Norway; 2 Department of Anesthesiology and Intensive Care, Stavanger University Hospital, Stavanger, Norway; 3 Faculty of Health Sciences, University of Stavanger, Stavanger, Norway; 4 Pre-hospital Division, Air Ambulance Department, Oslo University Hospital, Oslo, Norway; 5 Department of Emergency Medicine and Pre-Hospital Services, St. Olav’s Hospital, Trondheim, Norway; 6 Department of Anesthesiology, Vestfold Hospital Trust, Tønsberg, Norway; 7 Prehospital Division, Vestfold Hospital Trust, Tønsberg, Norway; 8 Norwegian Trauma Registry, Oslo University Hospital, Oslo, Norway

**Keywords:** Critical care, Comorbidity, Emergency medical services, Pre-hospital emergency care, Physicians

## Abstract

**Background:**

Individualized treatment is a common principle in hospitals. Treatment decisions are made based on the patient’s condition, including comorbidities. This principle is equally relevant out-of-hospital. Furthermore, comorbidity is an important risk-adjustment factor when evaluating pre-hospital interventions and may aid therapeutic decisions and triage. The American Society of Anesthesiologists Physical Status (ASA-PS) classification system is included in templates for reporting data in physician-staffed pre-hospital emergency medical services (p-EMS) but whether an adequate full pre-event ASA-PS can be assessed by pre-hospital physicians remains unknown. We aimed to explore whether pre-hospital physicians can score an adequate pre-event ASA-PS with the information available on-scene.

**Methods:**

The study was an inter-rater reliability study consisting of two steps. Pre-event ASA-PS scores made by pre- and in-hospital physicians were compared. Pre-hospital physicians did not have access to patient records and scores were based on information obtainable on-scene. In-hospital physicians used the complete patient record (Step 1). To assess inter-rater reliability between pre- and in-hospital physicians when given equal amounts of information, pre-hospital physicians also assigned pre-event ASA-PS for 20 of the included patients by using the complete patient records (Step 2). Inter-rater reliability was analyzed using quadratic weighted Cohen’s kappa (κ_w_).

**Results:**

For most scores (82%) inter-rater reliability between pre-and in-hospital physicians were moderate to substantial (κ_w_ 0,47-0,89). Inter-rater reliability was higher among the in-hospital physicians (κ_w_ 0,77 to 0.85). When all physicians had access to the same information, κ_w_ increased (κ_w_ 0,65 to 0,93).

**Conclusions:**

Pre-hospital physicians can score an adequate pre-event ASA-PS on-scene for most patients. To further increase inter-rater reliability, we recommend access to the full patient journal on-scene. We recommend application of the full ASA-PS classification system for reporting of comorbidity in p-EMS.

## Background

Tailored treatment through adapted choice of therapy, medication and monitoring to each patient is a common principle in hospitals [1–3]. In all parts of critical care, decisions are made based on the patient’s condition, including the patient’s comorbidities [1, 2, 4]. Decisions of dose adjusted medication and volume loading before anesthesia are common examples of individualized adaptions in the operating room [4]. Pre-hospital critical care is a continuum, and pre-hospital management is often a part of the patient’s course [5, 6]. As such, stratification on comorbidity, and individualized treatment, is equally relevant and valid for pre-hospital patients. In line with this principle, the patient’s health status before the acute event should be accounted for in triage on-scene and to determine threshold for, and timing of interventions and physiological targets [7, 8].

Risk adjustments allows for better judgement about the effectiveness and quality of alternative therapies [1]. Comorbidity is an important risk adjustment factor when evaluating pre-hospital interventions [9, 10]. In general, there is an agreement that outcome after trauma is influenced by the patient’s physical state before the trauma occurs [11]. Thus, to include a comorbidity measure is a prerequisite for comparisons and improves the precision of outcome prediction for trauma patients [8, 9, 12]. However, to obtain information on comorbidity from in-hospital records may be challenging for pre-hospital services due to logistics and legal issues of access and other strategies for obtaining this information should be explored.

Several methods for reporting comorbidities in pre-hospital emergency medical services (p-EMS) exists [8, 9, 13]. The American Society of Anesthesiologists Physical Scale (ASA-PS) classification system is used globally by anesthesiologists and classifies the preoperative physical health condition in patients before anesthesia and surgery. ASA-PS was originally designed to allow for statistical analyses of outcomes and to standardize terminology [14, 15], not to predict perioperative risk [15], but research has shown that the ASA-PS correlates well with overall surgical mortality [14]. Although the reliability of ASA-PS may be discussed, the scale is widely accepted as a tool to decide pre-operative health status [16]. The use of ASA-PS has expanded to the pre- and in-hospital critical care environment and pre-event ASA-PS, which is ASA-PS before the present injury or illness, [17] describes the inherent physiological state of a patient before an event. Pre-event ASA-PS is shown to be an independent predictor of mortality after trauma [8] and is included in templates for reporting of comorbidity in p-EMS and trauma [18, 19]. We therefore used pre-event ASA-PS as a comorbidity measure for the present study.

Ideally, pre-hospital services should have access to the full patient journal on-scene. Reality is however different and access to the full patient journal tends to be restricted for most pre-hospital services on-scene. P-EMS services must thus commonly base their decisions on the more limited amount of data and observations obtainable on-scene than for in-hospital physicians. Obtaining the complete medical history from seriously ill or injured patients on-scene is considered unfeasible, and reporting a dichotomized pre-event ASA-PS (pre-event ASA-PS 1 or pre-event ASA-PS > 1) is thus often recommended [20]. This simplification of the scale provides a very rough measure of comorbidity with low clinical discriminatory capabilities. Whether an adequate full pre-event ASA-PS can be assessed by pre-hospital physicians based only on the limited information generally available on-scene has not been explored and remains unknown. If scores between pre-and in-hospital physicians do not differ more than between in-hospital physicians, then the pre-hospital scores are just as “correct” as the in-hospital scores and can be used accordingly.

The aim of the present study was to explore whether it is possible for pre-hospital physicians to score an adequate pre-event ASA-PS already while on-scene.

## Methods

Prospective observational inter-rater reliability study. We assessed the degree of agreement among two raters using the ASA-PS scale under different circumstances to decide whether different access to information influenced the scores. All patients admitted by p-EMS to two Norwegian hospitals during a period of three-months (Stavanger University Hospital 19 Aug – 18 Nov 2016 and St. Olav University Hospital 1 Feb – 30 Apr 2017) were included. Following the inclusion periods, in-hospital physicians scored all included patients (Step 1). Data collection for the second part of the study (Step 2) was finished 21 Mar 2018. All Norwegian p-EMS services are staffed with anesthesiologists and respond to all types of emergency conditions, search and rescue missions and inter-hospital transfers.

We used the pre-event ASA-PS to assess comorbidity. The pre-event ASA-PS does not take the present event into account and describes the physiological state of the patient before an event [8, 11, 21]. The ASA-PS provides a global, subjective index of a patient’s overall health status, and pre-existing medical conditions are categorized on a scale of increasing medical severity (ASA-PS 1–5) [17].

### Step 1. Inter-rater reliability study of pre- versus in-hospital scores

Pre-hospital physicians assigned a pre-event ASA-PS score on-scene based on information available out-of-hospital only. The pre-hospital physicians did not have access to the full patient records. If the physician was unable to decide on a pre-event ASA-PS score on-scene, the score was kept unassigned and the main reason declared. After the three-month inclusion period, three in-hospital anesthesiologists at each of the two sites were given access to full patient records for all included patients at each site. Blinded from the pre-event ASA-PS score allocated by p-EMS each in-hospital physician used this information to assign pre-event ASA-PS scores for the included patients. No specific training for ASA-PS scoring was provided.

### Step 2. Inter-rater reliability with equal access to data

Because p-EMS generally do not have access to the full patient journal comparing pre-hospital on-scene scores with in-hospital scores is an asymmetric comparison (as in-hospital physicians have access to more information). We thus did not expect perfect agreement between pre- and in-hospital raters. To assess agreement of pre-event ASA-PS scores when pre- and in-hospital physicians had access to equal data, 20 patients were selected by an on-line randomizer and re-scored by the pre-hospital physicians when given access to complete patient records. The rationale behind this was to assess whether an observed difference in scoring was due to different physicians (pre- versus in-hospital) or different data availability.

We were unable to identify any studies in which pre-event ASA-PS was scored in a real-time pre-hospital setting. Without prior empirical information on the variation of the phenomenon under study we were consequently unable to perform sample size calculations [22, 23]. Statistical rules of thumb for sample size varies in the literature and sample sizes from 10 to 50 is reported [24]. Combining existing advice, we chose to included 20 patients per physician to evaluate inter-rater reliability [24]. If no agreement between pre- and in-hospital physicians for 20 patients could be established, we considered the pre-hospital scores to be irrelevant.

Patients and physicians were anonymized prior to further statistical analyses.

Guidelines for Reporting Reliability and Agreement Studies (GRRAS) was used [25].

### Statistical analyses

ASA-PS is an ordinal scale and agreement between two ASA-PS measures on the same individual was thus assessed using quadratic weighted Cohen’s Kappa (κ_w_); a modification of Cohen’s Kappa that also accounts for the degree of disagreement between raters [26]. κ_w_ is a number between 0 and 1. κ_w_ < 0.10 indicates no inter-rater reliability, while 0.11–0.40 indicates slight, 0.41–0.60 indicates fair, 0.61–0.80 indicates moderate and 0.8–1.0 indicates substantial inter-rater reliability [27].

If two measurement methods are to be considered similar their results should be indistinguishable from one another [28]. Using κ_w_ values between pre- and in-hospital physicians as a measure of agreement, we performed minimax hierarchical agglomerative clustering; a method for exploring the inner agreement structure of a dataset [29]. The result from this clustering process is presented visually as dendrograms. Such dendrograms look like up-side-down trees, grouping elements that agree the most near the bottom of the graph, with decreasing agreement (i.e. inter-rater reliability) the higher on the graph. This approach allowed us to visually explore whether the agreement between pre-and in-hospital physicians were indeed indistinguishable from one another. The overall mean agreement [30] for all pre- versus in-hospital physicians was also calculated. Data were analyzed using IBM SPSS statistics version 22 and R 3.1.0.

## Results

Pre-event ASA-PS was registered for a total of 312 patients. We excluded four patients admitted to non-participating hospitals and three patients without identifiable patient records. One physician scored only four patients, three with pre-event ASA-PS 3 and one that could not be scored. This did not allow for κ_w_ calculations, as scores were identical, and this physician and corresponding patients were thus excluded. In total 301 patients were available for further statistical analysis.

Pre-hospital physicians scored a median (range) of 21 (5–40) patients. Five patients (2%) could not be scored on-scene (four were unconscious and one was not able to communicate).

The distribution of ASA-PS scores between pre- and in-hospital physicians are presented in Table 1.

**Table 1:** Distribution of ASA-PS scores. Table depicts corresponding ASA-PS scores for pre- versus in-hospital physicians for each patient

	ASA-PS	1	2	3	4	5	Total
Pre-hospital scores	1	198	72	11	0	0	281
	2	34	211	110	4	0	359
	3	0	24	123	43	1	191
	4	0	2	18	13	0	33
	5	0	0	0	0	0	0
	Total	232	309	262	60	1	864
		In-hospital scores					

**Fig. 1:**
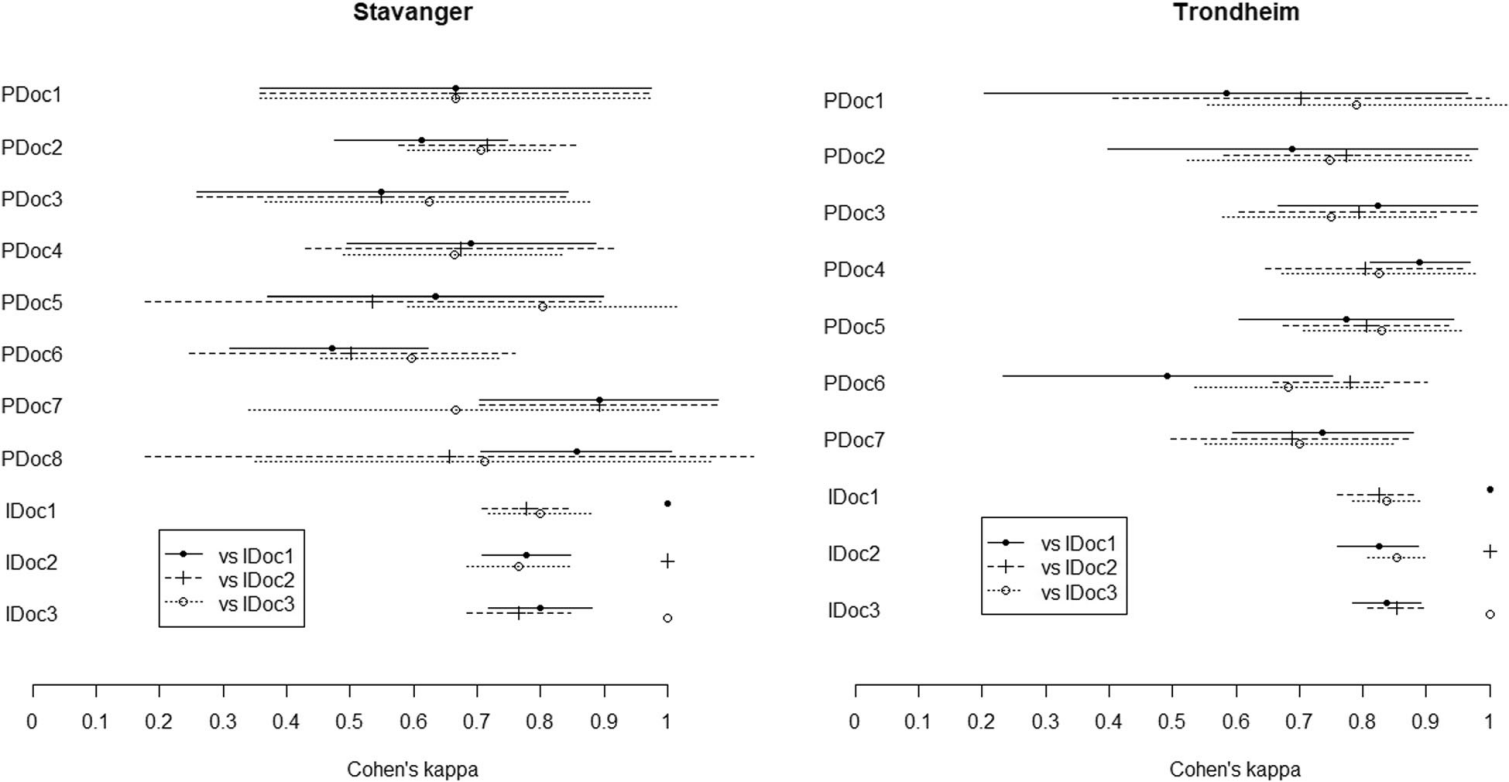
κ_w_ values for pre-event ASA-PS scores. Estimated inter-rater reliability between each pre-hospital (PDoc) and in-hospital (IDoc) physician using quadratic weighted Cohen’s kappa with 95% CI values

κ_w_ values for pre-event ASA-PS scores assigned by pre-hospital physicians on-scene, and subsequent scores based on complete patient records by in-hospital physicians are presented in Fig. 1.

κ_w_ values ranged from 0.77 to 0.85 among the three in-hospital physicians, and from 0.47 to 0.89 when comparing the pre- to in-hospital physicians. The mean kappa values were 0,67 (PDocs Stavanger), 0,78 (IDocs Stavanger), 0,75 (PDocs Trondheim) and 0,84 (IDocs Trondheim). For most scores (82%) inter-rater reliability between pre-and in-hospital physicians were moderate to substantial (κ_w_ > 0.61).

**Fig. 2:**
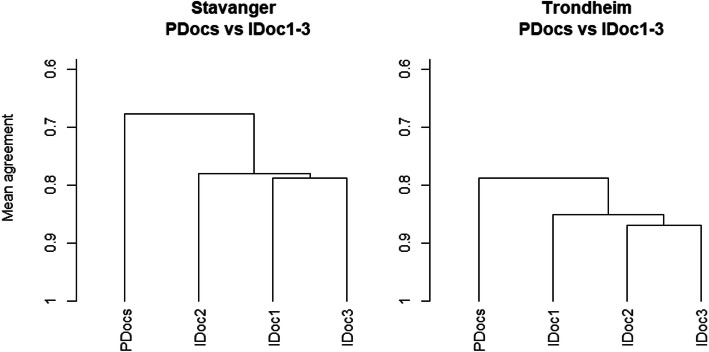
Pre- versus in-hospital agreement. Mean agreement between all pre-hospital physicians (PDocs) and the three in-hospital physicians (IDoc) at the two sites, using on-scene pre-hospital scores and in-hospital scores respectively

The mean agreement between all pre-hospital physicians and each of the three in-hospital physicians is generally high. However, the three in-hospital physicians tend to agree more with one another than they agree with the pre-hospital physicians. This is demonstrated in Fig. 2.

**Fig. 3:**
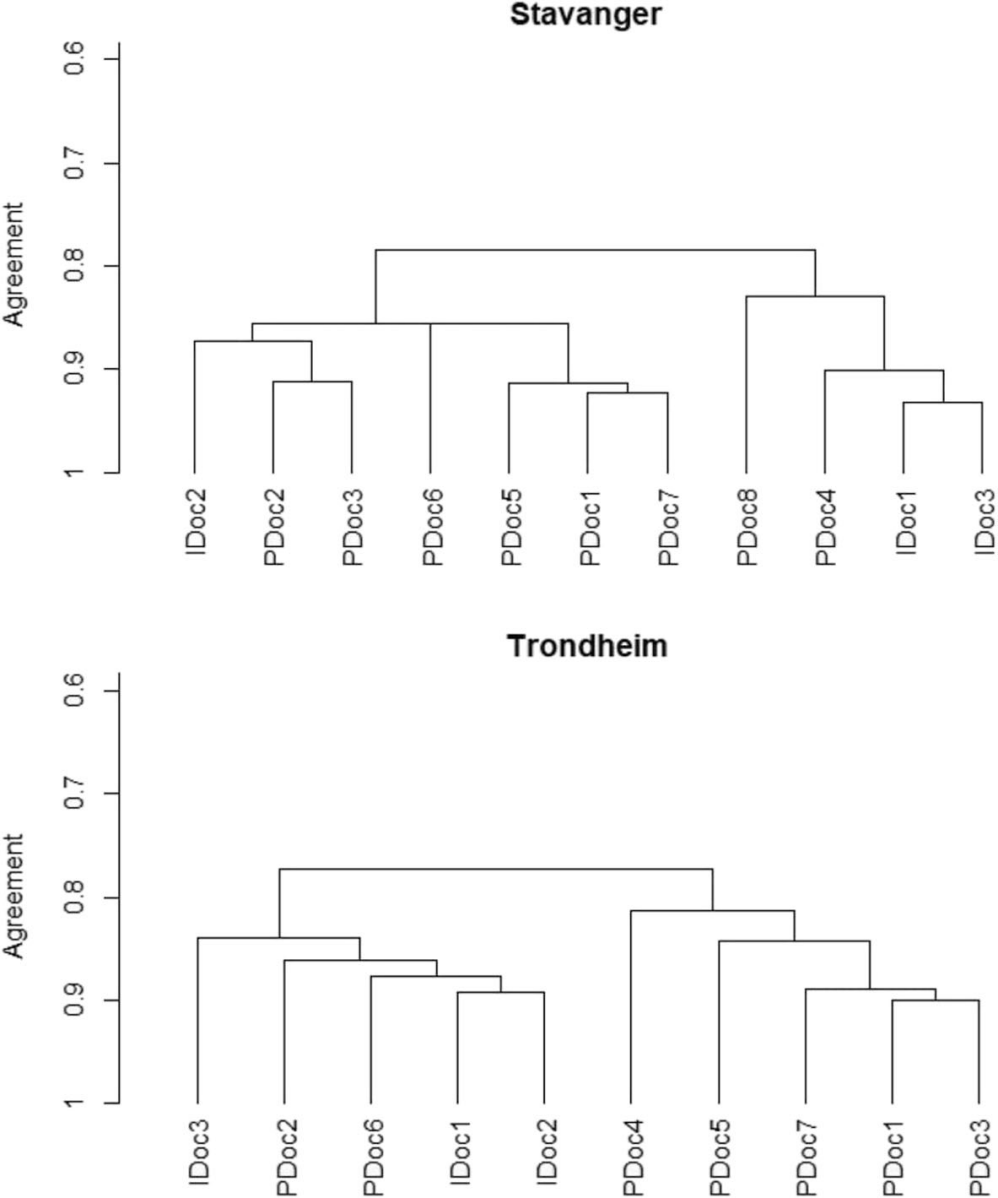
Agreement when given equal access to information. Dendrograms depict inter-rater reliability between pre- (PDoc) and in-hospital (IDoc) physicians when scoring the same 20 patients with pre-event ASA-PS given equal access to information. PDocs are indistinguishable from IDocs

When pre- and in-hospital physicians scored the same 20 patients with equal access to information, the agreement was strengthened. The difference in inter-rater reliability between the pre- and in-hospital physicians was much smaller, with κ_w_ values ranging from 0.65 to 0.93, indicating moderate to substantial agreement. Corresponding dendrograms for the two sites demonstrate that scores from pre- and in-hospital physicians do not cluster but remain largely indistinguishable from one another (Fig. 3).

## Discussion

The present study is a study of ASA-PS scoring in real life situations. As pre-hospital physicians did not have access to the full patient journal (Step 1), perfect agreement in ASA-PS scoring between pre-and in-hospital physicians was not to be expected. When comparing pre- and in-hospital pre-event ASA-PS scores, agreement was generally high ranging from fair to substantial. Most scores (82%) demonstrated moderate (64%) to substantial (18%) agreement, indicating that pre-hospital physicians can obtain sufficient data on-scene to score an adequate pre-event ASA-PS for most patients. Because the total number of pre-hospital scores are high, the impact of uncertainty in the scores, represented by broad 95% confidence intervals in Fig. 1, is reduced.

When pre- and in-hospital physicians scored pre-event ASA-PS on the same patients with access to complete patient records, agreement improved and ranged from moderate (52%) to substantial (48%). This indicates that ASA-PS scores from pre- and in-hospital physicians are indistinguishable from one another when they have equal data access (Fig. 3.). Accordingly, observed differences in pre-event ASA-PS scores in the first part of the study may be attributed to differences in data availability and time pressure on-scene rather than to factors related to individual physicians.

Comorbidity is an important risk-adjustment factor when evaluating pre-hospital interventions and the effect of p-EMS [9, 10]. Additionally, adjustment for comorbidity significantly increase the predictive accuracy of trauma outcome prediction models [9, 12, 31, 32]. The inherent nature of p-EMS favors a method for reporting comorbidities that is both readily available and time effective. ASA-PS is a well-known physical health condition scale, globally applied by anesthesiologists and surgeons, supporting the notion that pre-event ASA-PS may be advantageous for reporting comorbidity in p-EMS. However, studies have found substantial inter-observer variation [21, 33]. Most of these studies are hypothetical case scenarios designed by researchers [8, 16, 21]. In the present study we found that the agreement between pre- and in-hospital scores is acceptable for most patients and argue that pre-event pre-hospital ASA-PS should be applied for documentation of comorbidity in p-EMS.

Obtaining complete medical history from seriously ill patients on-scene is considered unfeasible. Accordingly, a dichotomized pre-event ASA-PS is often reported [20]. This is a very rough measure of comorbidity with low clinical discriminatory ability and will not distinguish between mild and severe systemic disease. Our results indicate that p-EMS can assign an adequate full-scale pre-event ASA-PS score already on-scene.

Significantly less accuracy of assigning ASA-PS is reported for non-anesthesiologists compared to anesthesiologists, possibly limiting the validity of pre-hospital pre-event ASA-PS scores to anesthesiologist-staffed services [34]. Standardized education and encouraged use may decrease variability for less proficient users [35]. Knowledge of comorbidity is relevant for all emergency medical services to aid decision-making and to target the treatment. Reliability of pre-event ASA-PS scored by paramedics is unknown and should be subject for further research. Precise definitions of each ASA-PS class, along with training for use, may improve reliability and usability for all users.

Although the physicians in the present study did not have access to patient records only 2% of the patients could not be scored on-scene, all of which had impaired consciousness. These patients remain a challenge for p-EMS regarding comorbidity assessment. Access to patient records in p-EMS may increase feasibility and precision of pre-event ASA-PS scores and systems for field data access should be available. Summary care records (SCRs) are electronic records of important patient information available for authorized health care staff involved in patient care [36]. The prevalence of summary care records (SCRs) is increasing [36]. SCRs may provide timely and relevant patient information regardless of regional affiliation. Whether access to SCRs will increase reliability of pre-event ASA-PS scores on-scene remains unknown.

### Limitations

The study was performed in a highly specialized anesthesiologist-staffed system and the results may not be transferable to other p-EMS. When number of assigned scores is low, conclusions may be inaccurate. Patients who died prior to hospital arrival were excluded. These patients are among the most severely sick or injured patients and may have a substantial comorbidity burden. Omitting these patients may overestimate the rate of agreement in this study.

## Conclusions

For an anesthesiologist-staffed EMS covering a mixed patient population, an adequate pre-event ASA-PS can be assigned on-scene. When data access was equal, pre-event ASA-PS scores by pre- and in-hospital physicians were indistinguishable from each other. When pre-event ASA-PS was scored on-scene with restricted data access, inter-rater reliability was lower, but acceptable. We recommend application of the full pre-event ASA-PS classification system for documentation of comorbidity in p-EMS.

## Data Availability

The datasets used and/or analyzed during the current study are available from the corresponding author on reasonable request.

## Abbreviations

ASA-PS: The American Society of Anesthesiologists Physical Status
p-EMS: Physician-staffed pre-hospital emergency medical services
GRRAS: Guidelines for Reporting Reliability and Agreement Studies
κ_w_: Quadratic weighted Cohen’s Kappa
PDoc: Pre-hospital physician
IDoc: In-hospital physician
SCRs: Summary care records
